# The Digital Therapeutics Real-World Evidence Framework: An Approach for Guiding Evidence-Based Digital Therapeutics Design, Development, Testing, and Monitoring

**DOI:** 10.2196/49208

**Published:** 2024-03-05

**Authors:** Meelim Kim, Kevin Patrick, Camille Nebeker, Job Godino, Spencer Stein, Predrag Klasnja, Olga Perski, Clare Viglione, Aaron Coleman, Eric Hekler

**Affiliations:** 1 Herbert Wertheim School of Public Health and Human Longevity Science University of California San Diego La Jolla, CA United States; 2 Department of Preventive Medicine Yonsei University College of Medicine Seoul Republic of Korea; 3 The Qualcomm Institute University of California San Diego La Jolla, CA United States; 4 The Design Lab University of California San Diego La Jolla, CA United States; 5 Laura Rodriguez Research Institute Family Health Centers of San Diego San Diego, CA United States; 6 Spiral Health Inc San Diego, CA United States; 7 School of Information University of Michigan Ann Arbor, MI United States; 8 Faculty of Social Sciences Tampere University Tampere Finland; 9 Small Steps Labs LLC dba Fitabase Inc San Diego, CA United States

**Keywords:** accessible, decision making, decision, decision-based evidence-making, development, digital therapeutics, medication adherence, monitoring, pharmaceuticals, public health, real-world data, real-world evidence, safe, testing, therapeutics

## Abstract

Digital therapeutics (DTx) are a promising way to provide safe, effective, accessible, sustainable, scalable, and equitable approaches to advance individual and population health. However, developing and deploying DTx is inherently complex in that DTx includes multiple interacting components, such as tools to support activities like medication adherence, health behavior goal-setting or self-monitoring, and algorithms that adapt the provision of these according to individual needs that may change over time. While myriad frameworks exist for different phases of DTx development, no single framework exists to guide evidence production for DTx across its full life cycle, from initial DTx development to long-term use. To fill this gap, we propose the DTx real-world evidence (RWE) framework as a pragmatic, iterative, milestone-driven approach for developing DTx. The DTx RWE framework is derived from the 4-phase development model used for behavioral interventions, but it includes key adaptations that are specific to the unique characteristics of DTx. To ensure the highest level of fidelity to the needs of users, the framework also incorporates real-world data (RWD) across the entire life cycle of DTx development and use. The DTx RWE framework is intended for any group interested in developing and deploying DTx in real-world contexts, including those in industry, health care, public health, and academia. Moreover, entities that fund research that supports the development of DTx and agencies that regulate DTx might find the DTx RWE framework useful as they endeavor to improve how DTxcan advance individual and population health.

## Introduction

Digital therapeutics (DTx) are health software tools designed to prevent, treat, or alleviate a disease, disorder, condition, or injury by delivering interventions that have demonstrable positive therapeutic effects on individual health and produce real-world outcomes [1-3]. DTx are often complex interventions [4] as they include multiple components, such as goal-setting or problem-solving elements, and algorithms that adapt the provision of support to each person’s changing needs. Common goals for DTx include improving medication adherence or regular use of medical devices (eg, glucometers), facilitating behavior change, such as improving diet, physical activity, or sleep, or improving mental health, such as care for depression, anxiety, or stress. DTx can also supplement other care, such as additional support in between clinic visits. The aspiration is that DTx can improve a patient’s health outcomes, reduce the burden on health care professionals, and increase access to and usability of interventions [1,5], by providing safe, effective, and equitable support for individual and population health [6]. While myriad frameworks exist for DTx development, to date, no single unifying framework guides DTx evidence production and regulatory decision-making [7-11]. By evidence production, we mean the use of scientific methods and processes to produce meaningful data about interventions, such as DTx, both qualitative and quantitative. By regulatory decision-making, we mean the set of oversight activities governing bodies such as the US Food and Drug Administration (FDA) engage in to ensure the products or services in a targeted sector (eg, the pharmaceutical industry) are safe, effective, and aligned with individual and societal needs. We believe that a framework that streamlines linkages between evidence production and regulatory decision-making for DTx will accelerate the development, adoption, and impact of DTx.

For a comprehensive framework to be successful, it must address 2 overarching issues that distinguish DTx from other therapeutics commonly used in health care. The first issue is that DTx are large pieces of software and thus benefit from decades of experience in how software is developed, used, and improved over time. The second issue relates to the relatively new regulatory environment for DTx, which has unique demands likely to evolve further as the field of DTx advances. With respect to the first issue, the basis of DTx in software renders them as dynamic entities that benefit from, and indeed require, periodic upgrades and regular maintenance to ensure they fit with ever-evolving user needs and technological changes. DTx needs to be interoperable with the constantly changing landscape of other software solutions used within health care and requires high levels of software sophistication based on enterprise-grade code embedded within a robust system architecture that supports security, privacy, and ongoing maintenance. Research-grade code is often of good enough quality to enable a novel digital health tool to be tested in small studies and efficacy trials, such as the activities commonly done by academics when engaging in frameworks like the Obesity-Related Behavioral Intervention Trials (ORBIT) [12], but it is rarely of sufficient quality to be sustainably deployed in real-world contexts. Thus, to be successful, a new framework must be able to guide appropriate evidence production that matches the inherent dynamic and often context-dependent nature of DTx.

The second overarching issue a new framework must address relates to regulatory issues. To date, regulation of DTx typically follows one of three approaches: (1) providing relatively limited guidance on evidence production, biasing toward the trustworthiness of DTx companies, as used in the FDA Precertification (Pre-Cert) Program [13]; (2) using emerging standards relevant to real-world data (RWD) and real-world evidence (RWE), such as reliance on data quality standards, use of RWD to efficiently run simulated clinical trials [14-17], and open science practices [18,19]; or (3) simply following variations of the 4-phase model [12] originally created for pharmaceuticals [20-22]. Payors are not providing adequate reimbursement systems, likely in part because of these issues, causing some DTx companies to declare bankruptcy [23]. We contend that a new framework that incorporates elements of these approaches may be helpful to multiple stakeholders.

## Our Approach

Based on this background, we had 2 primary objectives for our proposed DTx RWE framework (hereafter called the “Framework”): (1) to create for users a decision-focused flowchart of key steps to develop DTx, with clear go/no-go milestones needed to move between phases of DTx RWE production that maps to the needs of regulatory decision-making (a point we return to in the discussion); and (2) to provide guidance on how to use RWD to develop this RWE. The 4-phase model is adapted from the National Institutes of Health (NIH)–supported ORBIT [12] model for behavioral intervention development, with phases focused on design, development, testing, and monitoring. We also considered it important that the Framework provide guidance on RWD use and evidence production in accordance with safe, timely, effective, efficient, equitable, and patient-centered (STEEEP) targets [24], as well as accessibility [25], sustainability [26], and scalability [27].

To accomplish this, we synthesized frameworks and best practice methods from relevant fields including, but not limited to, behavioral medicine, psychology, public health, medicine, human-centered design, human-computer interaction, bioinformatics, agile software development, computer engineering, health equity research, and community-based participatory research. We drew upon a range of different scoping reviews of frameworks for DTx evidence production (eg, Torous et al [28] and Lagan et al [29]). However, rather than using a scoping review process or other formalized expert consensus approach, first and foremost, we were guided by the issues summarized above because we consider them critical to the development of successful DTx yet underemphasized to date.

While we drew from many sources, the ORBIT model, an NIH-recognized approach for behavioral intervention development that uses 4 phases analogous to pharmaceutical development, was a foundational source. However, for our purposes, the ORBIT model had important limitations. It is set up to be broad and accommodate the development and testing of a wide range of different types of behavioral interventions. This domain-specificity can make it challenging for those who may have limited familiarity with behavioral sciences to know how to use it for their specific needs. Additionally, the ORBIT model focuses on evidence production in support of the design of phase III efficacy trials. This is well-matched to studying novel interventions or the efficacy of technologies in ideal conditions, but it is very different from our goal of optimizing the development and sustainable deployment of DTx over time.

We also drew from expert consensus recommendations, including those from the World Health Organization (WHO) [30] and consensus statements from relevant workshops hosted by the NIH [31] within the United States, when creating the Framework. When necessary, the authors used first-hand knowledge based on their participation in expert consensus statements in related fields [7,32,33]; experience with both the research methods and community practices delineated in the Framework [8,32,34]; experience teaching graduate-level methods courses on topics covered in the Framework*;* and as innovators engaged in the development, use, and evaluation of novel methods explicitly created for digital health evidence production [35-37].

In addition, we drew on the FDA Pre-Cert Program, which was created to assess the credibility and readiness of a group to engage in DTx evidence production. The FDA Pre-Cert Program begins with an excellence appraisal, which aims to establish credibility by demonstrating the company’s readiness through evaluating organizational excellence and a culture of quality [13]. Following that, the product goes through a streamlined review process to ensure a reasonable level of safety and effectiveness assurance, which leads to a decision on whether commercial distribution is approved. Once the product is on the market, they are asked to provide RWE based on RWD with a limited list of clinical trial designs in a specified period of time. The Pre-Cert Program ensures that companies have high standards of organizational excellence, that they carry out real-world monitoring of the software as it is used, and, critically, provide a mechanism that could be used to allow DTx groups to be reimbursed in some fashion while RWE production occurs.

## The DTx RWE Framework

### Overview

**Figure 1:**
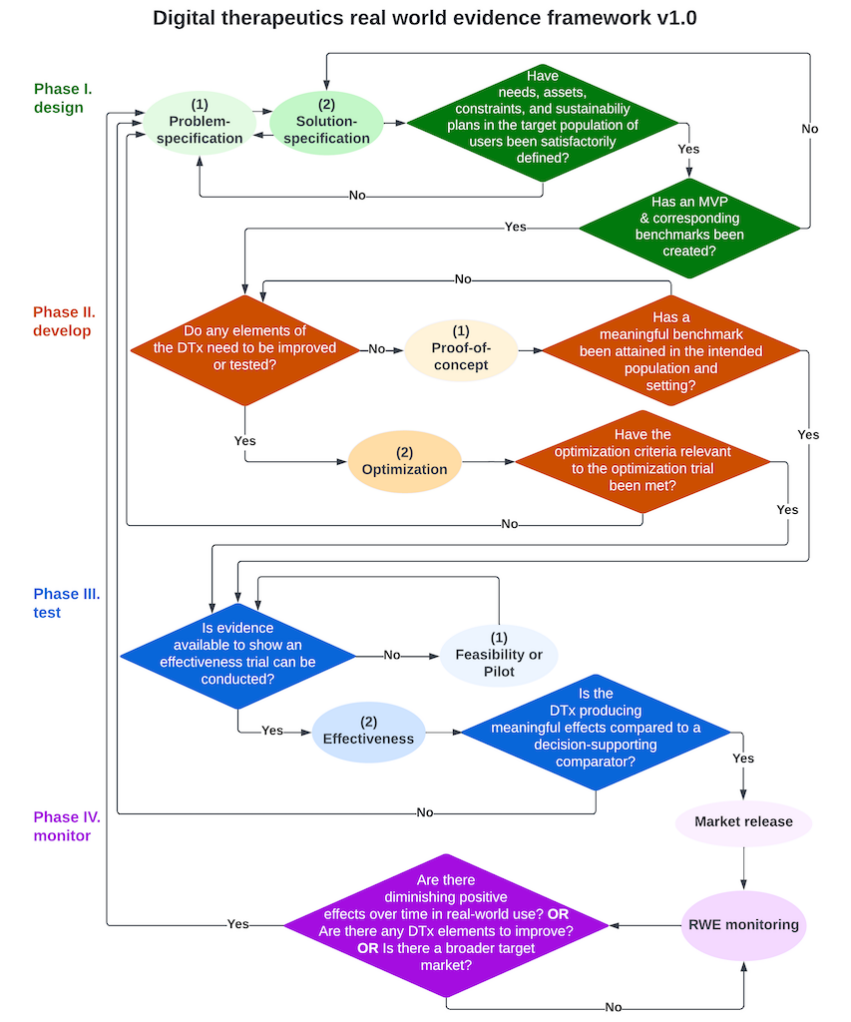
Digital therapeutics real-world evidence framework flowchart. Practical, iterative, and milestone-driven guidance to producing real-world evidence (RWE) during the design, development, testing, and monitoring phases of digital therapeutics (DTx). Oval shapes represent research or operation activities and diamond shapes represent decisions or review activities. MVP: minimal viable product.

The Framework (Figure 1) is centered on a flowchart with 4 phases analogous to the ORBIT model but adapted for DTx RWE production (phase I: design; phase II: develop; phase III: test; and phase IV: monitor). Phase I activities correspond to the “double diamond” approach [38] used in human-centered design and related methods where the problem and solution specifications are delineated. Phase II activities are drawn primarily from ORBIT [12], its extensions [39], and the Multiphase Optimization Strategy (MOST) [40,41]. Phase III activities are based on insights from pragmatic clinical trial best practices, including Pragmatic Explanatory Continuum Indicator Summary-2 (PRECIS-2) [42], reach effectiveness, adoption, implementation, and maintenance (RE-AIM) [43,44], and recommendations from an NIH-recognized expert panel on comparator selection for behavioral interventions [45]. Phase IV activities are drawn from implementation science [46,47], and emerging recommendations on RWE use for postmarketing surveillance from the WHO [48]. The approach to RWD in the Framework draws on recent recommendations for the use of RWD for pharmaceuticals [48-51] and on recommendations on open science best practices, which are integrated into each phase, with additional suggested recommendations [52] summarized in the Discussion section.

The Framework has 4 phases as described in detail below. Sufficient resources need to be provided at both the start of this process and as it goes through the development life cycle if it is to be successful. Moreover, analogous to what we outlined above for the FDA Pre-Cert process, we recommend that groups undertaking this process commit to the following, either on their own or through one or more partnerships, as illustrated in the Framework use case provided in Multimedia Appendix 1. With this, there are 3 critical roles that must be present:

1. Designate a DTx implementor with the capability to provide ongoing, sustained deployment of the DTx. Examples could be industry, medical centers, or public health departments with proven software development and management capabilities.
2. Designate a community-serving organization that is working with and serving a target population that can provide RWD. These could be hospitals and clinics, federally qualified health centers, community clinics, or public health departments.
3. Designate a DTx evaluator with expertise in the relevant methods and approaches recommended throughout the Framework, both in terms of the flowchart of research activities and the use of RWD.

The responsibility of key stakeholders across all phases of the Framework is to intentionally consider the relevant ethical, legal, and social implications of the DTx pipeline. Including a consultant on the team who is well versed in thinking about, for example, participant characteristics and enrollment, data management (eg, collection, storage, analysis, and sharing), and related issues of bias and privacy are important throughout the process in the Framework. Engaging with an ethics review board, like a research ethics committee or institutional review board, can also be useful at various points in the process.

### Phase I: Design

#### Overview

The goal of phase I is to design the DTx product. The two activities work iteratively together: (1) problem-specification and (2) solution-specification, in accordance with the well-recognized “double diamond” used in human-centered design [38]. Problem specification includes delineating real-world needs, constraints, assets and approaches to support future sustainability, both financial and ecological [53,54]. Solution specification focuses on iteratively creating a DTx through agile development and prototyping from low-fidelity (eg, paper concepts and storyboards) to high-fidelity (fully functional code) prototypes, with this iterative work being situated within ethical practices and exploring the potential for reusing components or functionalities from available DTx where possible, in line with open science practices [55].

Intervention plausibility claims are the key focus of phases I and II. We are explicitly using the word “plausible” instead of “feasible” given emerging nomenclature recommendations. By “intervention plausibility,” we are referring to context-dependent probabilistic claims regarding the interaction between the DTx and targeted populations and settings, such as acceptability, demand, capacity to be adapted to a local context, etc. (a full list of plausibility targets is available at [56]; note: it was labeled feasibility in this paper given that it was published before emerging nomenclature recommendations). Progress can be determined in phase I through the specification of benchmarks that conform with the specific, measurable, actionable, realistic, and timely (SMART) goal concept [57] but are applied to establishing benchmarks. The transition from phase I to II is justifiable when benchmarks relevant to, at a minimum, safety, and effectiveness are defined and a minimal viable product (MVP) DTx has been produced that meets basic ethical, functional, and usability requirements.

#### Question 1a: Have Needs, Assets, Constraints, and Sustainability Plans in the Target Population of Users Been Satisfactorily Defined?

This work specifies the target population and setting and answers other key questions relevant to DTx [7]. Foundational to this is the RWD from a community-serving organization to identify unmet needs, current assets (eg, standard practices and billable activities related to the targeted DTx), and constraints (eg, likely number of billable sessions and the scope of real-world needs, if scaled). Beyond RWD, a mixed methods approach is recommended for this stage. This includes formative research such as ethnographic studies, focus groups, and interviews; community-based participatory and community-driven methods; literature reviews, such as reviewing previous intervention and epidemiological studies; and market research and analysis. Determination of whether these have been satisfactorily defined can be achieved by reaching a consensus to determine if there is overarching agreement among stakeholders, which involves a decision being made when no one objects [58].

#### Question 1b: Have an MVP and Corresponding Benchmarks Been Created?

The focus is to iteratively build the DTx and finalize benchmarks. In terms of DTx creation, movement to phase II is justified when a group has produced evidence showing that an MVP is functioning according to minimal usability and accessibility requirements and meets a threshold of being plausible as a tool to enable phase II activities. This could be demonstrated through DTx that are free of “bugs” and meet basic usability requirements (eg, good System Usability Score [59]). Ideally, benchmarks are established in relation to STEEEP targets [24] as well as accessibility [25], sustainability [26], and scalability [27]. To guide future implementation, data could be gathered about critical implementation issues [56]. Minimal benchmarks are needed for safety and effectiveness.

When setting benchmarks, the team should balance what is meaningful relative to current best practices with what is plausible to achieve for the target population and setting [60]. These benchmarks should, ideally, be based on RWD and establish a threshold that defines if the proposed DTx could plausibly produce desired effects safely relative to current practices. Examples of benchmarks include “a decrease of 3% in hemoglobin A_1c_ among 60% of our DTx users after 6 months of intervention” for effectiveness and “less than 10% of our DTx users experienced nonserious adverse events associated with digital treatment after a month of intervention” for safety. With a functioning MVP and benchmarks defined, the team has produced the requisite information to transition to phase II. Key approaches here include agile or lean development practices, prototype testing and development, rapid prototype testing, and qualitative methods [61]. See Multimedia Appendix 1 for an example.

### Phase II: Develop

#### Overview

The goal of phase II is to guide DTx development and optimization. There are two types of activities: (1) proof-of-concept trials and (2) optimization trials. Movement from phase II to phase III requires RWE—produced either from a proof-of-concept trial or optimization trial—that demonstrates it is plausible that the DTx can produce clinically meaningful effects in the targeted population and setting while meeting requisite implementation requirements. Given that ORBIT is our primary starting point, it is important to flag that we are rearranging where these trials take place. Specifically, in ORBIT, optimization trials occur late in phase I (ie, phase Ib). In contrast, proof-of-concept trials occur in phase IIa in ORBIT. In the authors’ opinion, the approach used in ORBIT creates 2 issues. First, it places many important activities as phase I. Second, it implicitly signals that phase I and II activities are subservient to phase III activities (a point we return to when discussing phase III of the Framework). In the Framework*,* we unpack activities in phase I of ORBIT to spread them across phases I and II and provide more explicit labels of the key purposes for each phase, namely design and development. With this, our intent was to provide clearer guidance on how one progresses between phases and also to connote the unique value of each phase without any need for one to be subservient to another; instead, the phase is selected based on the type of evidence production needed.

Within this phase, RWD should be used to support targeted recruitment and selection of study participants with a particular eye toward accounting for health equity in defining a target population (eg, targeting a population that explicitly underuses the current standard of care). In addition, RWD can be used to monitor for unintended consequences, both positive and negative, of the use of the DTx within the development trials. RWD could also be used, particularly with deployed DTx, for conducting data-driven algorithm development [62,63].

#### Question 2a: Do Any Elements of the DTx Need to Be Improved or Tested?

The proof-of-concept trial focuses primarily on intervention plausibility testing about if the overall package is producing meaningful results, as defined through the benchmarks established in phase I. Optimization trials produce evidence to improve elements of the DTx. For example, optimization trials can be used to support the evidence-based selection of DTx components (factorial trials) [64-68], refinement of components, particularly those used across time (microrandomized trials) [35,69,70], and refinement of adaptation algorithms to match the provision of support to context, individual differences, and timing (microrandomized trials [35,71], and sequential multiple assignment randomized trials [72-75]). Note also that optimization trials could also be conducted that are explicitly used to support algorithm development (eg, system identification experiments [76-78] or more data-driven algorithm development from RWD [79,80]). Like phase I, proof-of-concept and optimization trials can be used iteratively. Determining if optimization is needed is based on whether any element of a DTx needs to be improved. If no elements of the DTx package need to be refined, then a proof-of-concept trial is appropriate. If some element of the DTx package needs to be tested or improved, then an optimization trial is needed.

#### Question 2b: Has a Meaningful Benchmark Been Attained in the Intended Population and Setting?

This is a fundamental question for the proof-of-concept trial, an emerging approach in behavioral trials that tests a DTx in a small group (eg, 10-20) in relation to a benchmark (eg, 70% of the 10 patients shift from hypertensive to systolic blood pressure <120 mm Hg). This approach is used because of 2 known issues with small sample trials within formative work. First, small samples render the use of frequentist inferential statistics problematic [60,81,82]. Second, humans have confirmation bias, which refers to the tendency for individuals to seek out or interpret evidence in ways that align with previous beliefs, expectations, or hopes [83]. A proof-of-concept trial overcomes these challenges without the need for running larger trials, using a clearly specified a priori benchmark that can be tested using descriptive statistics. Its use of benchmarks enables resource-efficient studies with clear go/no go decision-making that reduces the risk of falling prey to confirmatory bias. Quasi-experimental, single-case, or within-participant designs in which the participants serve as their own controls are appropriate design options for a proof-of-concept trial. Further, mixed methods, where both qualitative and quantitative data are relevant to the goals of evidence production and real-world intervention implementation plausibility [56] targets, should be used. Proof-of-concept trials provide clear go/no go milestones established a priori, thus reducing the risk of continuing when not justified, which is common with more traditional piloting study approaches [39]. If benchmarks are met, the group can either shift to an optimization trial or transition to phase III. If the benchmarks are not met, then the team should consider returning to phase I or focusing on DTx optimization.

#### Question 2c: Have the Optimization Criteria Relevant to the Optimization Trial Been Met?

Optimization is a concept drawn from engineering that emphasizes data-driven improvement for a DTx [41,84]. Optimization supports any problem arising with the DTx, such as the DTx costing too much, not being sufficiently adhered to, being difficult to implement in real-world contexts, being inaccessible to the target population, etc. Like a benchmark, the key logic here is to specify clear optimization criteria, meaning a definition of success that can be tested using an appropriately selected optimization trial. Specification of optimization criteria is a central focus of MOST [41,84]. The goal is to create optimization criteria that are measurable and, ideally, account for real-world constraints. For example, one could establish optimization criteria that a DTx includes only intervention components with demonstrated effectiveness, that the DTx can be deployed for under US $50 per client, or that total interactions per week with the DTx stay below 30 minutes. These criteria can be translated into clear go/no go criteria that can be assessed in an optimization trial. If the optimization criteria are met, then this can often justify movement to phase III. Plausible optimization trials for this phase could include but are not limited to: A/B testing (as used in the technology industry for improving usability) [85-87], factorial trials as used in MOST [64,65,67,68], sequential multiple assigned randomized trials [72-75], microrandomized trials [35,36,69-71], system identification experiments [76-78], studies explicitly designed to support algorithm development [79,80], and control optimization trials [37,88]. Nahum-Shani et al [40] provide guidance on when to use common optimization trial designs. See Multimedia Appendix 1 for an example.

### Phase III: Test

#### Overview

The goal of phase III is to test if the DTx produces meaningful improvements relative to a comparator in real-world contexts. There may be two types of activities: (1) feasibility or pilot studies; or (2) an effectiveness trial. As in phase II, we have shifted phase labeling from the original ORBIT model while still honoring the types of evidence production ORBIT generally advocates for. Specifically, in the ORBIT model, feasibility or pilot studies are conducted in phase IIb. In ORBIT, phase III is reserved purely for an efficacy trial to test if the intervention impacts health outcomes. We relabeled each phase intentionally in the Framework to allow each phase of work to be conducted and produce insights that are valuable alone, with no phase treated as subservient to other phases. Thus, we sought to have development phase activities stand alone in terms of their unique value for evidence production. We recommend this shift in thinking to clearly flag the critical, independent importance of each phase of work, particularly phase II development, which could feasibly be used in perpetuity alone as a rigorous approach to continuous quality improvement. While speculative, we contend that ORBIT and related evidence production models that implicitly or explicitly treat earlier phases as subservient to phase III trials, particularly efficacy trials in ideal conditions, send a message that privileges one type of evidence at the expense of other evidence. This is problematic, as the evidence from the other phases is particularly important for fostering real-world implementation and health equity. Further, privileging one type of evidence over others establishes the risk of a mono-method bias within scientific knowledge. This creates issues with fostering trustworthy scientific knowledge [89], reducing confidence in any consensus statements from overemphasizing one particular type of evidence, and might slow the pace of learning and progress [90]. This is particularly true when evidence production privileges tests occurring in ideal conditions, which is a valuable focus for novel interventions. When groups are developing novel interventions, they should use the ORBIT model, given its emphasis on ensuring appropriate evidence is produced to complete a high-quality efficacy trial. With phase III of the Framework, our focus is on testing if a DTx package produces meaningful results in real-world contexts to foster evidence production with high ecological validity. Thus, in the Framework we bias toward pragmatism through a focus on effectiveness trials, inclusion of benchmarks added to a modified CONSORT (Consolidated Standards of Reporting Trials) diagram guided by RE-AIM [91] to justify generalization claims, use of decision-oriented comparator selection, and emerging best practices on power calculations [39]. Moreover, the use of hybrid clinical trials, which incorporate the focus on testing both effectiveness and implementation outcomes [92,93] can be another option to be used in phase III testing of the Framework.

Even within the context of RWE, we recognize the classic tension between pragmatism and explanatory knowledge, as illustrated in PRECIS-2 [42] (eg, recruitment, eligibility, and setting). With context-invariant interventions, such as vaccines, explanatory knowledge tends to be highly valuable for producing both robust internal validity and generalizable knowledge. Further, with highly novel interventions, explanatory knowledge is valuable to determine if a signal is present in ideal conditions for the novel intervention. For evidence production to guide regulation of use in the real-world context of DTx, we suggest a bias toward pragmaticism over explanatory knowledge to support ecologically valid knowledge as an approach to increase the likelihood of generalizable knowledge. Recognizing that, just like in traditional trials, this tension must be balanced in each trial conducted based on the goals of the work and what is already known. RWD should be used to support recruitment, with a particular eye toward accounting for health equity in recruiting truly representative samples. Confidence in any claims of representation and, thus, generalizability, from the trial can be supported by clearly defining meaningful while also achievable benchmarks for reach and adoption, which can be measured and reported in a modified CONSORT diagram [91]. Furthermore, once a DTx is widely deployed, RWD could be used to run simulated clinical trials to test effectiveness in real-world contexts [14-17].

#### Question 3a: Is Evidence Available to Show an Effectiveness Trial Can Be Conducted?

This is a key question for feasibility or pilot studies, which are used to pave the way for a future effectiveness trial. A feasibility study examines whether and how a proposed or planned effectiveness trial can be done, but without a requirement for resemblance to the future trial [39]. Note, we are explicitly using the word “feasibility” only to refer to attributes of a targeted future fully powered trial, in alignment with 2010 CONSORT recommendations on clinical trial nomenclature [94,95]. The word “feasibility” is sometimes used in reference to issues about the intervention, such as if it would be acceptable, have sufficient demand, or could be integrated into real-world contexts [56]. To avoid confusion, we refer to these targets as intervention plausibility. Emerging recommendations from scientific groups, such as the 2010 CONSORT recommendations and others more relevant to digital health [96,97], suggest that the term “feasibility” be used to describe the probability that a particular type of study, such as a phase III clinical trial, can be conducted with sufficient rigor and fidelity by an investigative team in a given context. Based on this, we will honor this emerging consensus and only use the term “feasibility” to refer to context-dependent probabilistic claims in relation to the likelihood that a targeted study can be conducted in a particular setting by a particular group with sufficient fidelity to allow conclusions to be drawn from it. Similarly, we use emerging naming conventions and reserve the term “pilot” to refer to a specific type of feasibility trial that implements the exact eventual full protocol of a fully powered trial, but with fewer participants. The goal of a pilot study, thus, is to gather information about the likelihood that if a full trial were conducted, the data quality would be sufficient to enable trustworthy inferences [39]. If the evidence is available to show the feasibility of an effectiveness trial, the team can proceed directly to the effectiveness trial. If this evidence is not available, then the team should consider conducting a feasibility or pilot study before effectiveness trials to increase the likelihood that, if a trial is run, it will be conducted with sufficient fidelity to provide sufficient quality evidence to guide decisions.

#### Question 3b: Is the DTx Producing Meaningful Effects Compared to a Decision-Supporting Comparator?

The complexities of DTx establish higher evidentiary standards for generalization claims. By generalization, we specifically focus on transportability, meaning the degree to which insights gleaned from a given study sample are relevant to a stated population and setting. Recent studies of health behaviors, cognitive processes, and emotions, all factors that may influence the effectiveness of a DTx, show that behaviors, cognitions, and emotions have multiple contextual influences, can differ widely from person to person, and fluctuate over time within individuals [50,98-103]. Thus, research studies intended to produce generalizable knowledge about the effectiveness of DTx, including where, for whom, and when a given DTx is useful, need to take these factors into account. Given these complexities with regard to DTx evidence production, transportability is difficult to establish. We suggest, first, the use of a modified CONSORT diagram that integrates insights from the RE-AIM framework, and second, benchmarks be established relevant to the percentage of plausible settings and participants enrolled and completing the study. This modified CONSORT diagram provides an approach to quantifying the degree to which a sample may or may not be representative of a stated population and setting. If, for example, there is a large disparity between an eligible population and the number of patients who are enrolled, then transportability claims would be questionable. As with other phases in this Framework, it is suggested that achievable benchmarks related to the modified CONSORT diagram be specified a priori (eg, 80% of eligible clinics will take part, 80% of eligible staff will take part, and 50% of eligible participants will be enrolled). As before, these benchmarks need to balance the need to be ambitious while also being achievable based on what is known. Only if the benchmarks are met can transportability claims be justified. These benchmarks on the modified CONSORT diagram should be derived from RWD.

As this is an effectiveness trial, comparator selection should support real-world decision-making. For example, if the clinical or community partner has a current standard of care and they are considering replacing it with the DTx, the standard of care should be the comparator. Alternatively, if the DTx would fulfill a new area of need, a stepped wedge trial [104,105], in which the DTx is released in a phased fashion across clinics, could be considered. For detailed guidance on comparator selection, see NIH expert panel recommendations [45]. For DTx testing, options include but are not limited to between-person RCTs [106,107], including remote RCTs [108,109], cluster RCTs [110,111], stepped wedge trial [104,105], and, when sufficient RWD is available, the use of simulated clinical trials [14-17] could be considered.

We also recommend the use of best-practice recommendations for power calculations that specifically do not rely upon underpowered studies to infer effect sizes [60,112,113]. Instead, what is recommended is to establish 2 effect size estimates, a threshold of clinical significance, and a plausible effect size that could be observed in the trial. The threshold of clinical significance is the smallest effect size of interest [114] that would influence clinical decision-making based on an explicit qualitative determination of a noticeable difference. This threshold of clinical significance should be informed by RWD and can be translated from the benchmarks for effectiveness defined in phase I. The plausible effect size is the most likely effect size to be observed if the trial were conducted. This plausible effect size can be informed, in part, from the results of the proof-of-concept trial, particularly if the benchmark that was met is well matched to the threshold of clinical significance. That said, given the unreliability of small sample sizes, RWD and effect sizes from previous trials most like the proposed study should be used to establish the plausible effect size. If the plausible effect size is at or above the threshold of clinical significance, then a trial is warranted. If the plausible effect size is below the threshold of clinical significance, then the trial should not be conducted, as the results gathered would not be sufficient to make a convincing argument to change clinical practice.

If benchmarks set to the modified CONSORT diagram are not met or there is no clinically significant difference observed between the DTx and comparator, then returning to phase I or phase II activities is appropriate. If benchmark and clinically meaningful differences are observed, then results can be submitted to regulators for official review. If the trial was done ethically and responsibly and the results are positive, then regulating bodies can certify the DTx and allow the DTx to market to the population and setting that was studied within the phase III trial. See Multimedia Appendix 1 for an illustrative example.

### Phase IV: Monitor

#### Overview

The goal of phase IV is to monitor the use of the DTx within the real world, enable DTx implementers to improve the DTx with additional RWD collected from their clinical or community partners and support the expansion of the target market for the DTx through stepwise additional assessments. This is analogous to traditional non-DTx phase IV activities, including RWD use with pharmaceuticals [48].

#### Question 4: Are There Diminishing Positive Effects Over Time in Real-World Use? Are There Any DTx Elements to Improve? Is There a Broader Target Market?

As described earlier and elsewhere [7,32,115], continuous improvements in the DTx are not only desired but required for a DTx. For example, user interface expectations of technologies and the use of application programming interfaces drive the evolution of technology. A web application designed and tested in the 1990s [116,117], if it was not continually updated to meet changing user expectations and remain up-to-date with related application programming interfaces, would, at best, be perceived as “old” and, at worst, would not work. Thus, prespecified quality control methods for these updates would need to be used, and this could be one area where regulatory guidelines could be helpful. For example, there is active discussion about when the accumulative changes to a DTx warrant running another clinical trial [118].

Given this, any notion of a “definitive” clinical trial, a concept traditionally used, is inappropriate for DTx. Pragmatic ways of gleaning insights about when DTx is meeting expectations across the STEEEP and related criteria listed earlier are critical to monitor over time. To support this, the implementation of science practices, particularly strategies for ongoing monitoring, thoughtful adaptation, and guidance on rigorous continuous quality improvement, can be gleaned from the dynamic sustainability framework [46]. Through ongoing monitoring, issues of potential diminishing benefit (labeled voltage drop) can be observed and used to inspire a response [22]. For example, if effectiveness levels go below some predefined threshold, regulators could provide DTx with a time-limited window for continued marketing while also requiring the DTx company to reestablish a partnership with a clinical or community partner and reengage with earlier stages of the process. With this potential risk looming, it could establish an incentive for the DTx company to engage in continuous improvement, guided by the other 2 questions, and to maintain mutually beneficial partnerships.

These recommendations conform with recommendations from the WHO for monitoring and evaluating digital health interventions [119]. According to the WHO, the 4 major components of digital health monitoring (ie, functionality, stability, fidelity, and quality) should guide ongoing monitoring, with these questions mapping onto our proposed questions:

- Are there diminishing positive effects over time in real-world use?
  (Quality) Is the content and the delivery of the intervention of high enough quality to yield intended outcomes?
  (Quality) How well and consistently is the intervention delivered?
- Are there any DTx elements to improve?
  (Functionality) Does the system operate as intended?
  (Stability) Does the system consistently operate as intended?
- Is there a broader target market?
  (Fidelity) Do the realities of field implementation alter the functionality and stability of the system, changing the intervention from that which was intended?

RWE for postmarket surveillance is being explored, and opportunities and pitfalls that are also relevant to DTx are being articulated and should be considered regarding DTx regulation [48]. Monitoring could require benchmarks to be set for all key targets of evidence production (eg, effectiveness, safety, and equity) as one pathway for cultivating more rigor in monitoring efforts and reducing the risk of confirmation bias during phase IV.

If the DTx implementer believes their DTx can support a more diverse market share than what was approved in phase III, RWD collected in phase IV may help the DTx implementer accelerate this expansion. For example, monitoring could be used to identify plausible new populations, settings, or areas for improvement of the DTx, particularly if done with other community-serving organization partners who may have providers prescribing the DTx for “off-label” uses. One plausible way to improve evidence production during phase IV monitoring would be to focus evidence production more on testing and improving the elements of a DTx (eg, intervention components and adaptation algorithms) instead of the DTx package. A more detailed rationale for this is described elsewhere [7,32,47,82,120]. A second opportunity would be to link activities and efforts with ongoing behavioral ontology efforts to foster better knowledge comparison across various DTx [121,122]. With that said, standards for ongoing monitoring of RWD and RWE are rapidly evolving; thus, this is a critical area for continued work. See Multimedia Appendix 1 for an illustrative example.

## Discussion

### Overview

The Framework provides guidance to groups seeking to sustainably deploy DTx for use in real-world contexts and may be helpful to regulatory and funding entities as they provide support and oversight of DTx. We acknowledge that the Framework has not been rigorously vetted and that additional work is needed to establish its value. This includes determining whether the use of the Framework has greater or lesser utility in specific domains of DTx applications, such as those used in mental health, behavioral health, or as an adjunct to pharmacological and other interventions for chronic diseases like cancer, musculoskeletal disorders, and cardiovascular disease. We know of no specific reasons why such differences should exist, but as published reports of DTx research emerge in the future, these distinctions might become evident.

Another issue pertains to how the Framework can assist with evaluating DTx that are already in the field, including digital wellness tools that do not meet the definition of a DTx. The longer a DTx has been sustainably deployed at scale, the more likely it is that simulated clinical trial methods could be used to study DTx along STEEEP criteria. With this, efficiencies could be further advanced for evidence production through simulated clinical trials through RWD. Future work would benefit from continued focus on the refined development of simulated clinical trial best practices to improve the pace and resource efficiency of learning.

Regarding digital health wellness tools, these tools often build on foundational behavior change techniques, such as self-monitoring and goal setting that have decades of evidence to guide their design and implementation. When situations like this exist, the burden of proof should be to justify why current evidence is not sufficient already. The most likely gaps in research for these may relate to insufficient evidence for their effectiveness in a broad range of users or settings, so a targeted adaptation of the Framework to fill in these gaps might be the best approach. For example, future work could explore ways to adapt the Framework for use with community-based organizations and community-serving well-being institutions such as the YMCA or Jewish Family Services, along with corporate wellness and related wellness programs that are not implemented by or in partnership with the health care system.

With these limitations recognized, we expand below on the need for three areas of future work related to the Framework: (1) how using RWD advances health equity; (2) cultivating trustworthy partnerships that foster the use of Framework, as a secondary pathway to advance health equity; and (3) suggesting next steps with regard to regulation and funding.

### Advancing Health Equity Through RWD

#### Overview

In our view, increased sophistication on the effective use of RWD can become a critical tool to overcome some of the major challenges currently faced in health care, including identifying and addressing health disparities to advance health equity for all, and to foster more targeted and resource-efficient evidence production. RWD provides the information needed to specify unmet needs in general as well as those for individuals, communities, and populations where current practices are not producing desired results. This can help focus resource expenditures and efforts to reduce health disparities.

Future work could advance the use of RWD to drive the development of evidence-based solutions that serve communities most in need. Clinically meaningful benchmarks based on RWD provide an approach for guiding DTx development, both for individual DTxs and for DTxs at large. These would create pressure for DTx not simply to replicate existing standards of care but to improve upon them. Indeed, RWD can be used to establish benchmarks across the various evidence production targets, such as effectiveness, safety, and equity, to provide the foundational data needed to measure, monitor, and, thus, drive equitable progress in individual and population health.

#### Cultivating Trustworthy Partnerships

As presented in the section above introducing the Framework, we recommend a tripartite approach to its use, comprising an entity committed to sustaining the DTx, a community-serving organization from which the RWD comes, and an entity with appropriate expertise in RWE evaluation efforts. While these conditions can be met within well-resourced settings such as academic medical centers, we suggest that there are opportunities for implementing the Framework through partnerships among groups that may historically not have worked as closely together, such as industry partners working with federally qualified health centers and supported with an academic partner, as illustrated in Multimedia Appendix 1.

The trustworthiness of all actors involved must be acknowledged as a foundational starting point for any approach to evidence production [123,124]. This includes not merely thinking that trust can be achieved with effective communication but that, at its core, trust involves acknowledging and centering ethics, inclusion, and equity as central guiding principles in the work [89,123]. To do this, we propose the use of best practices in cultivating and maintaining partnerships that have already been delineated like community-based participatory research [125,126], patient-led innovation [127,128], community-driven design [129], community psychology practices [130,131], and ethical digital health research practices [33,34,132,133]. Incorporation of approaches to determining corporate trustworthiness that was formatively tested in the FDA Pre-Cert program can be used, including excellence appraisal, and streamlined review elements (eg, real-world performance plan and review determination information) [13]. The Digital Health Checklist [133] might also be helpful to guide ethical practices for evidence production relevant to DTx pertaining to issues such as accessibility, privacy, data management, balancing risks, and benefits, all grounded in fundamental ethical principles including respect for persons, beneficence, justice, and respect for law and the public interest.

#### Regulatory and Funding Issues

We encourage regulators and funders of DTx to explore whether the Framework can help guide their efforts. The principles embodied in the Framework could be used to establish generalized regulatory expectations for DTx. Clarifying these expectations could help get multiple DTx developers and purchases “on the same page” with respect to achieving and maintaining appropriate standards of quality throughout the life cycle of DTx use. Similarly, funders of DTx research and development, such as the NIH, the Agency for Healthcare Research and Quality, the Patient-Centered Outcomes Research Institute, and the Health Resources and Services Administration, could encourage applicants to use the Framework, and if they do, then demonstrate how they propose to achieve the benchmarks that it includes.

### Conclusion

The Framework is intended to improve evidence production and sustainable deployment of DTx in real-world contexts. The Framework provides guidance on how to design, develop, test, and monitor DTx, both in the early stages of their development and over time as they are used in real-world contexts. Our hope is that the Framework can help address issues commonly seen with DTx, including low DTx uptake, long-term sustainability, and insufficient attention to health disparities. Overall, there is considerable opportunity to improve individual and population health equitably through DTx, and we hope the Framework can contribute to this end.

## Appendix

Multimedia Appendix 1 The hypothetical example of DTx RWE Framework.

## Abbreviations

CONSORT: Consolidated Standards of Reporting Trials
DTx: digital therapeutics
FDA: Food and Drug Administration
MOST: Multiphase Optimization Strategy
MVP: minimal viable product
NIH: National Institutes of Health
ORBIT: Obesity-Related Behavioral Intervention Trials
Pre-Cert: Precertification
PRECIS-2: Pragmatic Explanatory Continuum Indicator Summary-2
RE-AIM: reach effectiveness, adoption, implementation, and maintenance
RWD: real-world data
RWE: real-world evidence
SMART: specific, measurable, actionable, realistic, and timely
STEEEP: safe, timely, effective, efficient, equitable, and patient-centered
WHO: World Health Organization
