# Supplementary material for: The Digital Therapeutics Real-World Evidence Framework: An Approach for Guiding Evidence-Based Digital Therapeutics Design, Development, Testing, and Monitoring
Source: J Med Internet Res. 2024 Mar 5;26:e49208. doi: 10.2196/49208 (PMC10951831; doi:10.2196/49208)
Supplement: Multimedia Appendix 1 [file jmir_v26i1e49208_app1.docx]

**Online Supplement**

This hypothetical example is based on an actual digital health company and healthcare organization, with co-authors from both (SS and JG respectively). The Digital Health start-up, Spiral Health, is seeking to become a DTx focused on providing treatment and prevention options for chronic musculoskeletal pain. Family Health Centers of San Diego (FHCSD), is the community-serving organization for the hypothetical example. FHCSD is one of the nation’s ten largest Federally Qualified Health Centers (FQHCs) operating in San Diego County. Note, in this hypothetical example, we do identify a third group, which we label as "Researchers." As flagged already, this role could be filled by academics or consulting groups, or researchers could be housed at either the community-serving organization or the DTx company. In this example, the role of researcher is being filled by an academic partner. As can be seen in the hypothetical example, we flag the FHCSD, as the community-serving organization, as the key initial driver as FHCSD as the RWD needed to specify where needs and opportunities for a DTx are most acute.

The following tables describe:

- [Table 1] Partnership Formation Discussion Points
- [Table 2] Phase I. Design Example
- [Table 3] Phase II. Develop Example
- [Table 4] Phase III. Test Example
- [Table 5] Phase IV. Monitor Example

**Table S1.** Partnership formation discussion points example.

|  | **Spiral Health** | **Family Health Centers of San Diego (FHCSD)** |
| --- | --- | --- |
| **Partnership building** | | |
| **Identify the Success Criteria** | 1) Patient-reported improvements in pain (e.g., VAS pain scale improvements) 2) Improvement in function as measured by the OLBPI 3) Increased self-efficacy in managing spine pain 4) High customer satisfaction 5) Develop a DTx that can be prescribed and reimbursed by insurance | 1) Provide everyone with caring, affordable, high-quality health care and supportive services;  2) Offer financially sustainable options to uninsured, low-income, and medically underserved populations. |
| **Boundaries of the Targeted Market** | Adults with chronic musculoskeletal pain, excluding those suffering from traumatic injury or cancer | People who are medically underserved, greatly impacted by SDoH (ie FQHC-eligible patients) |
|  | COMBINED: People who are medically underserved, impacted by SDoH (i.e., FQHC-eligible patients) and who suffer from chronic musculoskeletal pain | |
| **Overall Leadership Structure** | 1) **FHCSD** leads decision-making on defining real-world success criteria, constraints, and assets. Further, FHCSD, as an FQHC, has an ethical obligation to advance the health of their patients. Thus, FHCSD can play a key role in ensuring all ethical requirements (e.g., attaining appropriate institutional review from an external party) are conducted and maintained. With that said, all parties would be responsible for following ethical practices and principles.  2) **Spiral Health** leads decision-making on DTx development and solution specification that meets FHCSD success criteria within real-world constraints and assets and aligns with the purpose of Spiral's DTx offering. 3) **Researchers,** provides expertise and guidance on the appropriate and ethical use of methods relevant to each phase. Note that the role of Researcher can shift between phases as method requirements change between phases. Researchers can be internal staff at FHCSD or Spiral or could be drawn from external partners such as academic partners or consultancy firms. | |
| FHCSD=Family Health Center San Diego; VAS=Visual Analogues Scale; OLBPI=Oswestry Low Back Pain Index; SDoH=Social Determinants of Health; FQHC=Federally Qualified Health Center; DTx=Digital Therapeutics | | |

**Table S2.** Phase I: design example.

| **Phase I: Design** | |
| --- | --- |
| **Leadership Structure in Phase I** | 1) **FHCSD** is key driver of Phase I activities, supporting and organizing of patients, providers, and other stakeholders to define success, constraints, and assets. 2) **Spiral Health** engages, with the goal of specifying a solution that fits FHCSD's success criteria, constraints, and assets.  3) **Researchers**, facilitates team and partnership formation activities to facilitate robust ethical, shared decision-making and offers skills in human-centered design methods and processes. |
| **The Type of RWD to be used** | **Collect and organize relevant data from most similar standard practice to the DTx** (e.g., indicators effectiveness, attrition rates, safety risks, complications, rehospitalization rates, etc) from FHCSD's EMR, with a focus on carefully selecting a target population and setting, accounting for balancing the need to advance health equity (or at a minimum, not propagating health disparities) and the development and sustainment requirements of a DTx. |
| **Problem Specification** | **Methods used:** A series of mixed methods formative studies would be used to define problems, needs, assets, and constraints. This includes engaging in conducting data analytics on available RWD with a particular focus on identifying issues of health disparities, such as some populations not being served with current resources; needs such as some settings unable to offer in-person support; and benchmarks, such as current success rates for analogous interventions to Spiral Health among different populations. In addition, active use of qualitative methods, such as interviews, focus groups, field studies/contextual inquiry, diary studies, and direct observation, could be used among all relevant stakeholders including providers, patients, and hospital administrators, along with surveys to provide quantitative results. Finally literature reviews, both from academic and non-academic sources, would take place to help better specify the problem and also likely needs, assets, and constraints. Using an iterative approach focused on triangulating insights from disparate sources to a share understanding, the following problems, needs, assets, and constraints are identified.  **Problem:** FQHCs often lack or are limited in PT and chiropractic services compared to the need. Insurance based system often creates constraints on these services such as number of visits covered, expensive copays, and few actual minutes with a provider. Additionally, attending PT appointments can be inconvenient especially for those with transportation/time barriers.  **Needs:** - A convenient, effective, and low-cost solution to manage and reduce chronic musculoskeletal pain that could be billable within an FQHC and fit into current PT billability options.  **Assets:** - Current chronic pain management protocols.  - FQHC financial models for serving FQHC-eligible patients.  **Constraints:** - Patients' skepticism of the effectiveness of exercises for chronic musculoskeletal pain (negative prior experience with chronic musculoskeletal pain treatments and lack of results) - Patients having difficulty in behavioral/lifestyle changes required to commit time and energy towards consistent practice of therapeutic exercises - Different levels among patients' ability to use the technology in both assessments and following the instruction during the intervention - Clinician burnout.  - Limited clinician training in managing chronic musculoskeletal pain. - Insufficient physician buy-in. |
| **Solution Specification** | **Methods used:** Mixed methods would be used, but with the methods focus more on testing the solution. RWD is used, but, increasingly, to define target populations and settings, paying particular focus to seeking to produce DTx that are serving currently underserved populations and settings in the community and clearly specified benchmarks, in terms treatment response among the target population Spiral Health. In addition, literature reviews, both from academic and non-academic sources, would take place to find currently available tools and other resources that could inform the work. Qualitative methods are used such as co-design workshops, participatory design (e.g., the formation of a diverse team with representative of all key stakeholders working together over time to design solutions that fit their real-world needs(, think aloud usability testing, A/B testing, unmoderated testing of tools, such as formative studies to examine real-world use of a Spiral Health among all relevant stakeholders including providers, patients, and hospital administrators. Using an iterative approach focused on triangulating the design of a Spiral Health that is solving the problem identified in Phase I(a) accounting for needs, assets, and constraints, while also providing more details on key issues for creating a viable sustainable solution including a sustainability plan, intervention elements of Spiral Health, and implementation strategies that could enable the Spiral Health to be used in the community-serving organization, and Specific, Measurable, Actionable, Realistic, Timely (SMART) benchmarks, which, in this example would be:  **Solution:** Develop an effective, easy-to-use, affordable DTx for patients with chronic musculoskeletal pain who receive care from an FQHC.   **Sustainability Plan**  An approach to sustainably pay for Spiral Health services rendered within current billing constraints of an FQHC, should it be shown effective in Phase III within an FQHC population.   **The intervention elements of an MVP of Spiral Health** - Success story emails - Daily text reminders - Virtual Live Coaching, as a supplement to FHCSD-delivered care - Education about the patient’s body using the AI system, which helps explain the problem, what needs to change to see results, and how the exercises can make these improvements - Monthly tracking/retesting of functional movement scores to objectively show patient their progress - Clear instructional videos so the patient feels confident in performing the activities - Badges/points/rewards for daily engagement/completion of treatment plan  **Implementation elements needed to implement Spiral Health with FHCSD** - Create user's guide for both patients and clinicians to enhance the easy-of-use of the tool - Find the right balance between the effective and time management for treatment (e.g, provide 5 videos/day or customize the time for the patients - Add portals for clinicians to monitor patients' activities in managing their pain - Provide continuing education to the patients explaining the importance of ongoing exercise therapy - Collect the user experience feedbacks whether they would recommend to their friends/families  **Benchmarks** *Effectiveness* - Over 50% of enrolled patients of Spiral Health have improvement in 2 points in VAS Pain Scale **OR** the average of 15% improvement in OLBPI after using the 16 week program of Spiral Health. *Engagement* - Over 50% of enrolled users of Spiral Health have engaged in the program at least 5 days a week over the 16 week program. *Safety* - 100% of enrolled users experience no serious adverse events linked directly to use of Spiral Health Services. |
| **Go/No go to next phase decision:** | ***Question 1a:*** *Have needs, assets, constraints, and sustainability plans in the targeted population of users been satisfactorily defined?* ***→ Yes:*** *Move to Question 1b* ***→ No:*** *Move to Problem Specification in Phase I. Design* ***Question 1b:*** *Have benchmarks and, at minimum, an MVP version of the DTx been created?* ***→ Yes:*** *Move to Phase II. Develop* ***→ No:*** *Move to Solution Specification in Phase I. Design* |
| DTx = Digital Therapeutics; FHCSD=Family Health Center San Diego; RWD=Real-world Data; EMR=Electronic Medical Record; VAS=Visual Analogue Scale; OLBPI=Oswestry Low Back Pain Index; PT=Physical Therapy; FQHC=Federally Qualified Health Center; MVP=Minimal Viable Product | |

**Table S3.** Phase II: develop example.

| **Phase II: Develop** | |
| --- | --- |
| **Leadership Structure in Phase II** | 1) **Spiral Health** is the key driver of Phase II, in both developing and optimizing DTx to meet benchmarks. 2) **FHCSD** leads decision-making on defining benchmarks.  3) **Researchers** design and conduct a POC and/or Optimization Trial(s) |
| **The Type of RWD to be used** | **RWD** is used to specify and justify sufficient need for a **targeted population and targeted setting.** In this example, that translates to FHCSD patients eligible to work with a PT but not engaging in care. RWD also used to monitor for **unintended consequences** of use of Spiral Health, such as reduced used of other services, increases in pain reporting, hospitalizations, etc. |
| **Decision-making Scenario 1: *(Question 2a)* *Do any elements of the DTx need to be improved or tested?*** | SCENARIO 1:  FHCSD reviews Spiral Health plans and determines that 1) if Spiral Health existed, it would be an important and valuable addition to FHCSD's care offerings **AND** 2) the benchmarks are meaningful and could plausibly be met among patients at FHCSD. Spiral Health reviews DTx elements and determines it is: 1) feasible to implement all DTx elements with fidelity and without additional need for development, **AND** 2) that they are confident that all the elements are needed.  IF THE ABOVE IS TRUE THEN: **Researchers,** in partnership with Sprial Health and FHCSD, designs and conducts a POC at and with FHCSD. |
| **Proof-of-concept** | **Researchers** conduct a POC trial (N=10), testing if Spiral Health achieves the targeted benchmarks established in Phase I (see above), conducted at FHCSD, as an indicator of plausibility. Within POC, a mixed methods approach would be use that include gathering surveys and feedback to test assumptions on key implementation requirements such as sufficient acceptability, demand, capacity to be integrated into current work, etc. These mixed methods would incorporate feedback from all relevant stakeholders, particularly the targeted patient population, providers, and healthcare administrators. Prior to conducting the POC appropriate ethical review would take place, such as receiving approval from an Institutional Review Board. |
| **Decision-making Scenario 2: *(Question 2a)* *Do any elements of the DTx need to be improved or tested?*** | SCENARIO 2:  FHCSD reviews Spiral Health plans and determines that: 1) if Spiral Health produced the targeted effects, it would be an important and valuable addition to their care offerings **BUT** The benchmarks are meaningful but there is insufficient evidence to conclude that Spiral Health could achieve said targets with the currently available Spiral Health DTx package. Spiral Health reviews DTx elements and determines it is: 1) plausible to implement all of DTx elements at this time **BUT** 2) Spiral Health is *NOT* confident that all the elements are needed, particularly the 'live coaching', given the added costs and impact live coaching has on scalability potential.  IF THE ABOVE IS TRUE THEN: **Researchers,** in partnership with **Sprial Health** and **FHCSD,** designs and conducts a Optimization Trial at and with FHCSD. |
| **Optimization** | **Researchers** design and conduct a screening experiment/factorial as used in MOST, to produce evidence needed for guiding evidence-based optimization of the Spiral Health DTx. Within the optimization trial, a mixed methods approach would be use that include gathering surveys and feedback to test assumptions on key implementation requirements such as sufficient acceptability, demand, capacity to be integrated into current work, etc. These mixed methods would incorporate feedback from all relevant stakeholders, particularly the targeted patient population, providers, and healthcare administrators. Prior to conducting the POC appropriate ethical review would take place, such as receiving approval from an Institutional Review Board. Further, open science practices would be expected such as trial registration, open sharing of treatment protocols, and public sharing of data used for analyses, whenever possible.  **Optimization criteria set by Spiral Health and FHCSD for a factorial/screening experiment as used in MOST** 1) DTx only includes components shown to improve the effectiveness of the intervention **AND** total costs remain under the plausible billable amount. 2) Is it **plausible** that benchmarks (see Phase I) would be achieved if the optimized (based on results of the screening experiment) were implemented? |
| **Go/No go to next phase decision:** | ***Question 2b:*** *Has a meaningful benchmark been attained in the intended population and setting?* ***→ Yes:*** *Move to Phase III. Test* ***→ No:*** *Move to Question 2a* ***Question 2c****: Have the optimization criteria relevant to the optimization trial been met?* ***→ Yes:*** *Move to Phase III. Test* ***→ No:*** *Move to Problem Specification in Phase I. Design* |
| DTx=Digital Therapeutics; FHCSD=Family Health Center San Diego; RWD=Real-world Data; PT=Physical Therapy; POC=Proof-of-concept; MOST=Multiphase Optimization Strategy | |

**Table S4.** Phase III: test example.

| **Phase III: Test** | |
| --- | --- |
| **Leadership Structure in Phase III** | 1) **Researchers** design and conduct Feasibility/Pilot Trial and/or an Effectiveness Trial. 2) **FHCSD** decides the decision-supporting comparator and supports Researchers with conducting trial with real-world patients and access to EHR/RWD.  3) **Spiral Health** offers robust version of DTx for testing at FHCSD. |
| **The Type of RWD to be used** | RWD used to specify and justify the credibility of Spiral Health by gleaning data from 53 clinics with PT services within FHCSD and among those clinics, it is found that 80% of patients who receive care cane be used to specify a benchmarks for specifying target populations and specific clinics that will be targeted to reduce health disparities. |
| **Decision-making Scenario 1: *(Question 3a) Is evidence available to show an effectiveness trial can be conducted?*** | SCENARIO 1:  **Researchers,** in partnership with FHCSD and Spiral Health, review the available literature and results from the POC trial and determines there is **not yet sufficient evidence to confidently conduct a well-powered, minimal attrition, effectiveness trial at FHCSD**.   IF THE ABOVE IS TRUE THEN: **Researchers**, in partnership with **FHCSD** and **Spiral Health**, design and conduct a Feasibility study, at and with FHCSD. |
| **Trial Feasibility or Pilot* Study** | - **Researchers,** in partnership with **FHCSD** and **Spiral Health**, create a protocol for a full effectiveness trial, and then **pilot tests the full effective trial protocol in a pilot study** with 40 patients at FHCSD to gather data about how feasible it is to recruit, randomize, and retain participants at FHCSD. A mixed methods approach would be use that include gathering quantitative date on key trial feasibility parameters such as recruitment rates, attrition in the two groups, and survey results to gather data on levels of usability and acceptability of the intervention, as well as qualitative results to examine issues such as fidelity of delivering both the intervention and control condition, perceived utility, and to identify and potential issues not captured with quantified data, including to qualify any quantitative results, such as insights about factors that impact recruitment rates. These mixed methods would incorporate feedback from all stakeholders, particularly the targeted patient population, providers, and healthcare administrators. Prior to conducting the study appropriate ethical review would take place, such as receiving approval from Institutional Review Board. |
| **Decision-making Scenario 2: *(Question 3a) Is evidence available to show an effectiveness trial can be conducted?*** | SCENARIO 2: **Researchers**, in partnership with **FHCSD** and **Spiral Health**, review the available literature and results from the proof of concept trial [or reviews results from a completed pilot trial, see Scenario 1] and determines there **is sufficient evidence to be confident that well-powered, minimal attrition, effectiveness trial can be conducted at FHCSD** and **that it is plausible that the intervention will produce a clinically meaningful effect that surpasses the TCS (defined via benchmark from Phase I).**  **Researchers**, in partnership with FHCSD and Spiral Health, conduct an Effectiveness Trial. |
| **Effectiveness** | **- Researchers**, in partnership with **FHCSD** and **Spiral Health** would conduct an effectiveness trial. A mixed methods approach would be use that include gathering quantitative data on key trial feasibility parameters such as recruitment rates, attrition in the two groups, and survey results to gather data on levels of usability and acceptability of the intervention, as well as qualitative results to example issues such as fidelity of delivering both the intervention and control condition, perceived utility, and to identify any potential issues not captured with quantified data, including to qualify any quantitative results, such as insights about factors that impact recruitment rates. These mixed methods would incorporate feedback from all relevant stakeholders, particularly the targeted patient population, providers, and healthcare administrators. Prior to conducting the effectiveness trial an appropriate ethical review would take place, such as receiving approval from an Institutional Review Board. At the completion of the trial, results will be shared with all relevant stakeholders both to return information and value to all stakeholders and to invite all stakeholders to provide feedback and clarify any proposed conclusions to be advanced when the trial is reviewed for regulatory approval. Further, open science practices would be expected such as trial registration, open sharing of treatment protocols, and public sharing of data used for analyses, whenever possible.  **Comparator** - Current standard of care for musculoskeletal pain is to work with a physical therapist. Within this effectiveness trial RCT, the comparison will be between standard of care PT support compared to an adapted standard of care that includes fewer PT visits (e.g., assessments, active monitoring) + Spiral Health.   **Power Calculations** TCS is defined as a 15% reduction in pain measured by the OLBPI scale compared to the standard of care. A statistician determined that this pain reduction corresponds to a TCS effect size of 0.4. Based on the percentage of individuals who achieved the target reduction or better, and the effects observed in similar interventions, the results of a previous POC trial (from Phase II) suggest that this level of pain reduction is plausible. Furthermore, based on the results of a pilot study and previous trials at FHCSD, the assumption for participant attrition is set at 30%. As a result, the final target sample size is 140 participants, plus an additional 60 to account for expected attrition. Thus, the total recruitment target is 200 participants.  **Benchmarks related to the modified CONSORT diagram (generalization claim tests)** - 80% of eligible staff involvement AND 80% of eligible FHCSD participants enrolled in the trial |
| **Go/No go to next phase decision:** | ***Question 3b:*** *Is the DTx producing meaningful effects compared to a decision-supporting comparator? → Yes: Move to Phase IV. Monitor → No: Move to Problem Specification in Phase I. Design* |
| DTx=Digital Therapeutics; FHCSD=Family Health Center San Diego; RWD=Real-world Data; PT=Physical Therapy; TCS=Threshold of Clinical Significance; RCT=Randomized Controlled Trial  *Note, a Pilot study is a specific type of feasibility, whereby the goal is to determine if the fully powered trial can be conducted with sufficient fidelity at the setting. Other types of feasibility trials can and should be used, following emerging guidelines and best practices on this work and approach. | |

**Table 5.** Phase IV: monitor example.

| **Phase IV: Monitor** | |
| --- | --- |
| **Leadership Structure in Phase IV** | 1) **Spiral Health** leads deployment and scaling to populations aligned with evidence produced from Phase III. 2) **Researchers** develop a RWE monitoring plan for detecting diminishing positive effects, elements to improve within DTx, or population expansion possibilities 3) **FHCSD** becomes a customer to Spiral Health, with Evaluator given access to RWD. |
| **The Type of RWD to be used** | Collect data to claim that Spiral Health **is living up to its promise in accordance with the benchmarks established in Phase I.** |
| **Target market release beyond partner** | Once benchmarks and clinically meaningful differences are observed, the DTx may be approved for market release to the targeted population and setting beyond FHCSD (partner). |
| **RWE monitoring *(if any of these examples apply, return to the activities from Phase I. Design)*** | **Methods used:** - Emerging data science practices for on-going monitoring would be devised and reviewed by Spiral Health, Researchers, and FHCSD. Further, open science practices would be expected such as use of data standards, best practices in data management and data informatics, open sharing of treatment protocols, and public sharing of data used for analyses, whenever possible. On-going data analytics would be run by Spiral Health, to monitor for diminishing positive effects as well as opportunities for improvement of the DTx or opportunities to broaden the target market. In addition, a plan for on-going communication (e.g., yearly reports developed by Spiral Health for FHCSD, researchers, and relevant stakeholders including targeted end-users) would be established to support on-going accountability and also to invite communication that could inspire iterative return back to earlier phases to improve upon Spiral Health. With this, the following would be explored:  **[Diminishing positive effects]** - If reduced health benefits are observed in RWD. If this happens, Spiral Health reverts to early phases with a clinical partner. Clinical partner could be FHCSD or new partner.   **[The improvements in DTx elements]** - Usage results reveal spots in the use of Spiral Health that could be improved. Spiral Health identifies the possibility for new technologies to be integrated (e.g., AR/VR systems) that could better support the Spiral Health. If this happens, Spiral Health reverts to early phases with a clinical partner. Clinical partner could be FHCSD or new partner.   **[A broader target market]** - Off-label uses of Spiral Health, prescribed by providers serving patients in targeted areas, suggest greater possible utility for Spiral Health. If this happens, Spiral Health reverts to early phases with a clinical partner. Clinical partner could be FHCSD or new partner. |
| FHCSD=Family Health Center San Diego; RWE=Real-world Evidence; RWD=Real-world Data; AR/VR=Augmented Reality/Virtual Reality | |
